# Supplementary material for: Genome variants associated with RNA splicing variations in bovine are extensively shared between tissues
Source: BMC Genomics. 2018 Jul 4;19:521. doi: 10.1186/s12864-018-4902-8 (PMC6032541; doi:10.1186/s12864-018-4902-8)
Supplement: Supplementary file 1 — Supplementary Methods. (DOCX 42 kb) [file 12864_2018_4902_MOESM1_ESM.docx]

**Supplementary Methods for ‘Genome variants associated with RNA splicing variation in bovine are extensively shared between tissues’**

*Sampling and tissue processing protocols for Experiment II and III*

**Experiment II**: Cows were firstly milked during routine morning milking, and again at 1 hour later, into a bucket. At each milking time point, up to 150ml of milk was sampled from the bucket and transferred to ice. Milk was then transferred to an on-farm laboratory where two 50ml aliquots of each milk sample were taken and 50ul of 0.5M EDTA added to each. Milk was then centrifuged at 1800rpm at 4 degrees celsius for 10 minutes. Milk fat was collected from top layer and skim milk in the middle layer decanted, leaving cell pellet. Multiple aliquots of 500ul of Milk fat added to 1ml of Trizol. Excess cream was wiped from the falcon tube with a little ethanol on a kimwipe. Cell pellet was then washed twice by adding 10ml PBS solution with 0.5mM EDTA and centrifuging for 10 minutes at 4^o^C. Supernatant was decanted leaving ~200ul. The cell pellet in the first falcon tube was resuspended in the remaining supernatant and transferred to the second falcon tube and the second cell pellet resuspended. The entire suspension was then transferred to a 2ml microcentrifuge tube, spun down, the supernatant pipetted off and then resuspended in 200ul of PBS solution and 1ml of RNAlater. After milk sampling the cows were individually restrained in a crush and given an intravenous injection of 10% zylazil adequate to cause moderate sedation. Each cow was then immediately released from the crush and upon the cow laying down a veterinarian euthanized the animal by lethal injection, using Pentabarb (sodium pentobarbitone 200mg/ml) administered intravenously at dose rates greater than 100mg/kg until the cow was deceased. Once pronounced dead mammary gland tissue was dissected from the mammary gland, just above the teat. Connective tissue was then removed and the samples dissected into 1cm squares, sealed in a 5ml tube and flash frozen in liquid nitrogen. All samples were then transferred to main laboratory on either ice (milk cells) or in liquid nitrogen canisters (mammary gland) and stored at -80^o^C.

**Experiment III:** Blood was collected by venipuncture of the coccygeal vein after routine morning milking and was processed according to the blood fractionation and white blood cell stabilisation procedure in the RiboPure™ blood kit (Ambion by Life Technologies) protocol. Milk samples of Holstein and Jersey cows were taken as described for experiment II at either Agriculture Victoria Research dairy herd (Holstein) or Wallacedale Jersey Stud (Jersey), both in Victoria, Australia.

*RNA sequencing protocols for Experiment II and III*

**Experiment II:** RNA was extracted from milk cells using Trizol Plus RNA Purification Kit (Ambion) according to manufacturer’s instructions. 100mg of mammary tissue was ground using a TissueLyserII (Qiagen) and liquid nitrogen. RNA was extracted from ~30mg of ground tissue using Trizol Plus RNA extraction kit (Invitrogen/Ambion) according to manufacturer’s instructions. RNA was then passed through an RNeasy column (Qiagen) and eluted in 30ul RNase free water. RNAseq libraries were prepared using the SureSelect Strand Specific RNA Library Prep Kit (Agilent) according to manufacturer’s instructions. Each library was uniquely barcoded, assigned to one of two pools and sequenced on a HiSeq™ 3000 (Illumina) in a 150 cycle paired end run. One hundred fifty base paired end reads were called with bcltofastq and output in fastq format.

**Experiment III:** RNA was extracted from milk cells using Trizol Plus RNA Purification Kit (Ambion) according to manufacturer’s instructions. RNA was extracted from white blood cells using RiboPure Blood Kit (Ambion) according to manufacturer’s instructions. All Holstein RNA samples had RNA integrity numbers greater than 6 for both milk cell and white blood cell RNA (in case of Holsteins). All Jersey milk cell RNA samples had RIN greater than 6. RNAseq libraries were prepared using the SureSelect Strand Specific RNA Library Prep Kit (Agilent) according to manufacturer’s instructions. Each library was uniquely barcoded randomly assigned to one of four pools and sequenced on a HiSeq™ 3000 (Illumina) in a 150 cycle paired end run. One hundred fifty base paired end reads were called with bcltofastq and output in fastq format.

*RNA sequencing data processing*

All data were assessed using FastQC. QualityTrim (https://bitbucket.org/arobinson/qualitytrim) was used to trim and filter poor quality bases and sequence reads. Adaptor sequences and bases with quality score <20 were removed. Reads with mean quality score less than 20, greater than 3 N, greater than three consecutive bases with quality score less than 15, or final length less than 50 bases were discarded. Paired RNA reads for each sample were aligned to the Ensembl UMD3.1 bovine genome assembly using TopHat2 [1] allowing for two mismatches. Custom computer scripts were used to assess sequencing performance, library quality and produced BAM file quality. The sequencing and QC parameters of experiment I can be found in [2] and for experiment II-IV the data were summarised in the below table:

| Experiments | Tissue | Read length (base) | Pair-end | Average raw reads (million) | Average reads Pass QC % | Average uniquely aligned reads (million) | Average concordant alignment rate % |
| --- | --- | --- | --- | --- | --- | --- | --- |
| II | Milk cells | 150 | YES | 46 | 79% | 33 | 90% |
| II | Mammary gland | 150 | YES | 98 | 82% | 74 | 91% |
| III | White blood cells | 150 | YES | 33 | 87% | 25 | 85% |
| III | Milk cells | 150 | YES | 72 | 91% | 57 | 87% |
| IV [3] | Liver | 100 | YES | 34 | 92% | 8.9 | 78% |
| IV [3] | Muscle | 100 | YES | 40 | 88% | 11 | 90% |

*RNA sequencing data quality check*

Qualimap 2 [4] was used to estimate read coverage and percentage of reads mapped to different origins of the bovine reference genome (UMD3.1). The default parameters of the RNA-seq QC function was used to analyse each tissue sample and the resulting data was averaged based on tissue type. The gene coverage uniformity of all genes and the 500 lowest and 500 highest expressed genes was obtained and averaged based on tissue types and standard errors calculated. The mean and standard errors were used to draw gene coverage uniformity plots in Supplementary Figure S1. The plots for all genes and highly expressed genes indicated little 5’ bias (Supplementary Figure S1a,b) and therefore little RNA degradation. Some 5’ bias was observed for genes that were lowly expressed (Supplementary Figure S1c). Means and standard errors of reads mapped to exonic, intronic and intergenic regions of the genome for each tissue types were also calculated as shown in Supplementary Table S1. The majority of the reads were mapped to exonic and intronic regions of the bovine reference genome. Up to 33% of the reads mapped to the intergenic regions.

*Splicing junction check of RNA sequencing data*

In the current study we used TopHat2 [1] to align transcriptomic assemblies. Given the existence and prevalence of other aligners such as HISAT2 [5] and STAR [6], we performed a small scale comparative analysis of alignments (BAM files) generated by TopHat2, HISAT2 and STAR. Since the current study was designed for RNA splicing, we focused on the mappable reads for splicing events and junctions that can be detected in BAM files, using RSeQC [7]. We randomly selected four samples, one white blood cell sample, one milk cell sample, one muscle sample and one liver sample, which represented four tissues types where the splicing sQTLs were mapped. The trimmed RNA seq data (fastq files) of these four tissue samples were re-aligned to Ensembl bovine UMD3.1genome assembly with HISAT2 and STAR. The new 8 BAM files (4 from HISAT2 and 4 from STAR) along with the 4 BAM files previously generated using TopHat2, were used for the junction analysis in RSeQC. Using the default setting of the junction_annotation function of RSeQC, the reads mapped to ‘Known Splicing Events’, ‘Known Splicing Junctions’, ‘Novel Splicing Events’, ‘Novel Splicing Junctions’, ‘Partial Novel Splicing Events’, ‘Partial Novel Splicing Junctions’, ‘Total splicing Events’ and ‘Total splicing Junctions’ were obtained and compiled in Supplementary Table S2. For these known categories, i.e., previously annotated in bovine genome, the differences of detectable splicing events and junctions were small between BAM files generated using different software. For ‘Known Splicing Events’ and ‘Known Splicing Junctions’ of the blood and milk samples, TopHat2 produced the highest estimates compared to HISAT2 and STAR. These results supported the adequacy of TopHat2 to detect known splice events and junctions. However, for all categories that are unknown, i.e., currently are not annotated in bovine reference genome, HISAT2 and STAR consistently produced higher estimates than TopHat2. These results suggest that HISAT2 and STAR are better at detecting novel splice events and junctions but also that a better genome annotation would improve splicing event and junction detection. Also, the read coverage appeared to strongly affect the result of splicing junction and event detection. White blood and milk cell tissues that had the longer read-coverage (150 PE) always showed many more detectable splicing junctions and events than liver and muscle tissues that had the lower read-length (100 PE) (Supplementary Table S2).

We performed a splicing junction saturation analysis for all tissues studied using RSeQC [7]. The analysis sampled subsets of BAM files from 5% to 100 % of mapped reads and then determined the mean number of reads mapped to splicing junctions for each subset. If the curve reached plateau prior to 100% then the dataset has likely detected the majority of splicing junctions. The results of the saturation analysis with the means and standard errors for each tissue types were calculated and are shown in Supplementary Figure S2. These results suggested that for all and known splicing junctions, the data appeared to saturate splicing junctions (Supplementary Figure S2a,b). There appeared to be more potential of splicing junction detections for the novel category (Supplementary Figure S2c), which could be improved with a better annotated reference genome or using HISAT2 or STAR for read alignment.

*Comparison between variants called from RNA-seq and from imputation*

The imputed whole genome sequence used in this study was imputed using Fimpute software [8] with Run5 of the 1000 bull genome project [9]. The average imputation accuracy of was 0.925 (0.898 ~ 0.952 depending on chromosomes) [10]. Theoretically, it may be useful to call variants from transcriptomic data and compare their genotypes (termed as ‘RNA sequence genotypes’) with the imputed sequence variant genotypes (termed as ‘imputed sequence genotypes’) to verify the imputation accuracy. However, in practise, the evaluation of imputed sequence genotypes using RNA sequence genotypes is complicated by several factors. Firstly, the sequencing errors originated from RNA-seq and imputed sequence data can both contribute to mismatches between the RNA sequence genotypes and imputed sequence genotypes. Secondly, gene expression can be tissue-specific and therefore an RNA sequence genotype cannot be determined from RNA sequence data from a tissue that does not express that gene. Thirdly, gene expression has been shown to be allele specific(ASE) in cattle [2], human [11] and mouse ([12]). ASE, results in an imbalance of the alleles expressed at any one SNP, at its most extreme it results in mono allelic expression, where only one of the two alleles is expressed. Such bias reduces the confidence in genotype calls from RNA sequence data, and in the case of mono allelic expression will result it incorrect genotype calling. For example, an imputed site that is heterozygous in a specific animal might be recorded as homozygous in the same animal for the RNA-seq genotype. This could arise as a result of ASE, where only one transcript with a specific allele at this genotype is expressed. Furthermore, if RNA-seq data from multiple tissues is available, ASE may occur in one tissue and not another, producing conflicting RNA-seq genotypes to match to imputed data. In short, a proper investigation of the consistency between the RNA sequence genotypes and imputed sequence genotypes will require a large amount of high quality RNA sequence data covering many tissue types and with prior knowledge of existing ASE so that these SNP could be masked in the comparison. This is clearly not the aim of the current study.

Nevertheless, as a proof of concept, we compared the imputed sequence genotypes with RNA sequence genotypes estimated from those animals with white blood cell RNA-seq data (Table 1) which appeared to have the highest quality in this study. For all variant sites detected in the 1000 bull genomes project [9] that were heterozygous in the animals with white blood cell RNA sequence data we estimated allele counts using Samtools mpileup [13]. In total 2,971,898 unique genome sites from the 1000 bull genome project can be detected across all animals combined with blood RNA-seq data and of these 69,449 are variants common to all animals. Since RNA-seq data can contain errors, we also restricted the selection of genome sites to those ones that had combined counts of reference and alternative alleles ≥ 10. This filter resulted in 24,407 variants remaining for comparisons between RNA sequence genotypes and imputed sequence genotypes.

For the imputed sequence data with the VCF format, the genotypes were coded as ‘0’ for reference allele homozygote (0|0 in VCF), ‘2’ for alternative allele homozygote (1|1 in VCF) and 1 for heterozygous (0|1 or 1|0 in VCF). In this VCF file the actual DNA base was also available for the reference and alternate alleles. In the RNA seq data, if the one or two allele types of the genome site were not those provided in the VCF as reference or alternate alleles, e.g, ‘A’ and ‘G’ in the RNA-seq but ‘C’ and ‘T’ in the imputed sequence VCF file, this genome site was given a genotype score of ‘3’ and defined as a case of ‘complete mismatch’. Else, if the genome site satisfied the following condition, (difference in the counts between the reference and alternative alleles) / (combined counts of the reference and alternative alleles) > 0.5, this genome site was defined as either a reference allele homozygous (‘0’) or an alternative allele homozygous (‘2’). Else, if the counts of both of the reference and alternative alleles >0, the genome site was defined as heterozygous (‘1’). For a genome site, if the genotype score of the imputed sequence (‘0’, ‘1’ or ‘2’) was different from the RNA-seq (‘0’, ‘1’, ‘2’ or ‘3’), and if the RNA-seq genotype score is a homozygous but the imputed sequence genotype score is heterozygous, this genome site was defined as ‘possible mismatch’. The term ‘possible mismatch’ was based on the fact that this type of mismatch may be associated with ASE or RNA-seq tissue specific information. The total number of mismatch was the combined number of ‘complete mismatch’ and ‘possible mismatch’. The proportion of matched cases, i.e., concordance, was 1-[(the total number of mismatch) / (total number of SNPs entered the analysis for each animal)].

**List of References**

1. Kim D, Pertea G, Trapnell C, Pimentel H, Kelley R, Salzberg SL: **TopHat2: accurate alignment of transcriptomes in the presence of insertions, deletions and gene fusions.** *Genome Biol* 2013, **14:**R36.

2. Chamberlain AJ, Vander Jagt CJ, Hayes BJ, Khansefid M, Marett LC, Millen CA, Nguyen TTT, Goddard ME: **Extensive variation between tissues in allele specific expression in an outbred mammal.** *BMC Genomics* 2015, **16:**993.

3. Khansefid M, Millen CA, Chen Y, Pryce JE, Chamberlain AJ, Vander Jagt CJ, Gondro C, Goddard ME: **Gene expression analysis of blood, liver, and muscle in cattle divergently selected for high and low residual feed intake.** *J Anim Sci* 2017, **95:**4764-4775.

4. Okonechnikov K, Conesa A, García-Alcalde F: **Qualimap 2: advanced multi-sample quality control for high-throughput sequencing data.** *Bioinformatics* 2015, **32:**292-294.

5. Kim D, Langmead B, Salzberg SL: **HISAT: a fast spliced aligner with low memory requirements.** *Nat Methods* 2015, **12:**357.

6. Dobin A, Davis CA, Schlesinger F, Drenkow J, Zaleski C, Jha S, Batut P, Chaisson M, Gingeras TR: **STAR: ultrafast universal RNA-seq aligner.** *Bioinformatics* 2013, **29:**15-21.

7. Wang L, Wang S, Li W: **RSeQC: quality control of RNA-seq experiments.** *Bioinformatics* 2012, **28:**2184-2185.

8. Sargolzaei M, Chesnais JP, Schenkel FS: **A new approach for efficient genotype imputation using information from relatives.** *BMC Genomics* 2014, **15:**478.

9. Daetwyler HD, Capitan A, Pausch H, Stothard P, Van Binsbergen R, Brøndum RF, Liao X, Djari A, Rodriguez SC, Grohs C: **Whole-genome sequencing of 234 bulls facilitates mapping of monogenic and complex traits in cattle.** *Nat Genet* 2014, **46:**858-865.

10. Pausch H, MacLeod IM, Fries R, Emmerling R, Bowman PJ, Daetwyler HD, Goddard ME: **Evaluation of the accuracy of imputed sequence variant genotypes and their utility for causal variant detection in cattle.** *Genet Sel Evol* 2017, **49:**24.

11. Consortium G: **Genetic effects on gene expression across human tissues.** *Nature* 2017, **550:**204.

12. Crowley JJ, Zhabotynsky V, Sun W, Huang S, Pakatci IK, Kim Y, Wang JR, Morgan AP, Calaway JD, Aylor DL, et al: **Analyses of allele-specific gene expression in highly divergent mouse crosses identifies pervasive allelic imbalance.** *Nat Genet* 2015, **47:**353-360.

13. Li H, Handsaker B, Wysoker A, Fennell T, Ruan J, Homer N, Marth G, Abecasis G, Durbin R: **The Sequence Alignment/Map format and SAMtools.** *Bioinformatics* 2009, **25:**2078-2079.
