# Supplementary material for: Genome variants associated with RNA splicing variations in bovine are extensively shared between tissues
Source: BMC Genomics. 2018 Jul 4;19:521. doi: 10.1186/s12864-018-4902-8 (PMC6032541; doi:10.1186/s12864-018-4902-8)
Supplement: Supplementary file 2 — Figure S1. Gene coverage uniformity plots for each tissue type for a) all genes, b) 500 highest expressed genes and c) 500 lowest expressed genes, as determined by Qualimap 2 [1]. Figure S2. Splicing junction saturation analysis for each tissue type for a) all splicing junctions, b) known splicing junctions and c) novel splicing junctions. Figure S3. The distribution of concordance between imputed and RNA sequence genotypes. Figure S4. Sample principal components clustering based on exon expression. Figure S5. Correlation of sQTLs (splicing quantitative trait loci), eeQTLs (exon expression QTLs) and geQTLs (gene expression QTLs) effects (t values) between tissues. Figure S6. Sharing of sQTLs (splicing quantitative trait loci), eeQTLs (exon expression QTLs) and geQTLs (gene expression QTLs) in paired tissues at p < 0.05 level. Figure S7. Manhattan plot of eeQTLs for the MGST1 gene. (PDF 737 kb) [file 12864_2018_4902_MOESM2_ESM.pdf]

Supplementary Figures S1-7 for:

Genome variants associated with RNA splicing variation in bovine are extensively shared between tissues

Ruidong Xiang[1,2], Ben J. Hayes[2,4], Christy J. Vander Jagt[2], Iona M. MacLeod[2], Majid Khansefid[2], Phil J. Bowman[2,6], Zehu Yuan[2], Claire P. Prowse-Wilkins[2], Coralie M. Reich[2], Brett A. Mason[2], Josie B. Garner[2], Leah C. Marett[2], Yizhou Chen[5], Sunduimijid Bolormaa[2], Hans D. Daetwyler[2,6], Amanda J. Chamberlain[2] and Michael E. Goddard[1,2]

[1]Faculty of Veterinary & Agricultural Science, University of Melbourne, Parkville, VIC 3010, Australia. [2]Agriculture Victoria, AgriBio, Centre for AgriBioscience, Bundoora, VIC 3083, Australia. [3]Agriculture Victoria, Dairy Production Science, Ellinbank, VIC, 3821, Australia. [4]Queensland Alliance for Agriculture and Food Innovation, Centre for Animal Science, University of Queensland, St. Lucia, QLD 4067, Australia. [5]Elizabeth Macarthur Agricultural Institute, New South Wales Department of Primary Industries, Camden, 2570 NSW, Australia. [6]School of Applied Systems Biology, La Trobe University, Bundoora, VIC 3083, Australia.

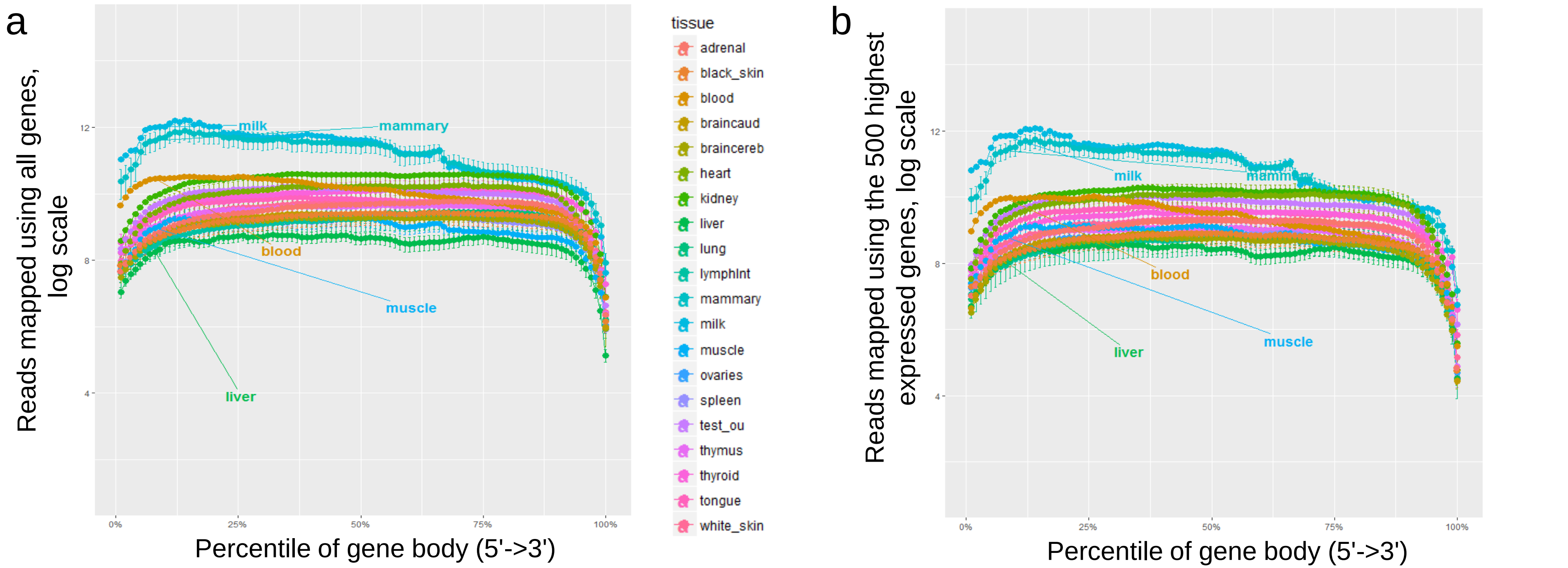

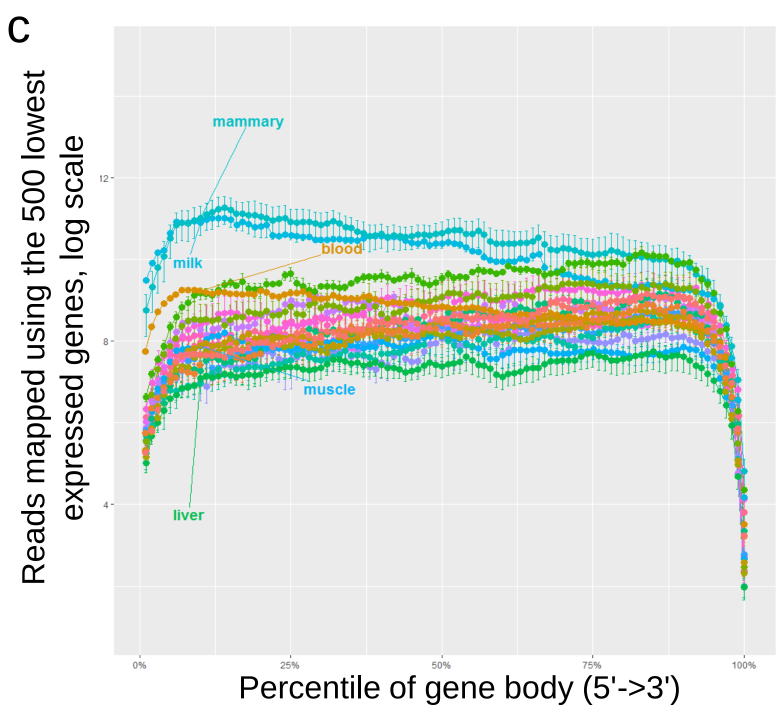

Figure S1. Gene coverage uniformity plots for each tissue type for **a**) all genes, **b**) 500 highest expressed genes and **c**) 500 lowest expressed genes, as determined by Qualimap 2 [1]. These plots show the mean number of reads mapped (y-axis) ,with standard error bars, to the length of the gene body (x-axis), where the length is expressed as a percentile of total gene length. braincaud: brain caudal lobe. braincereb: brain cerebellum. lymphInt: Intestinal lymph node.

[1] Okonechnikov, K., Conesa, A., and García-Alcalde, F. (2015). Qualimap 2: advanced multi-sample quality control for high-throughput sequencing data. *Bioinformatics* 32(2), 292-294.

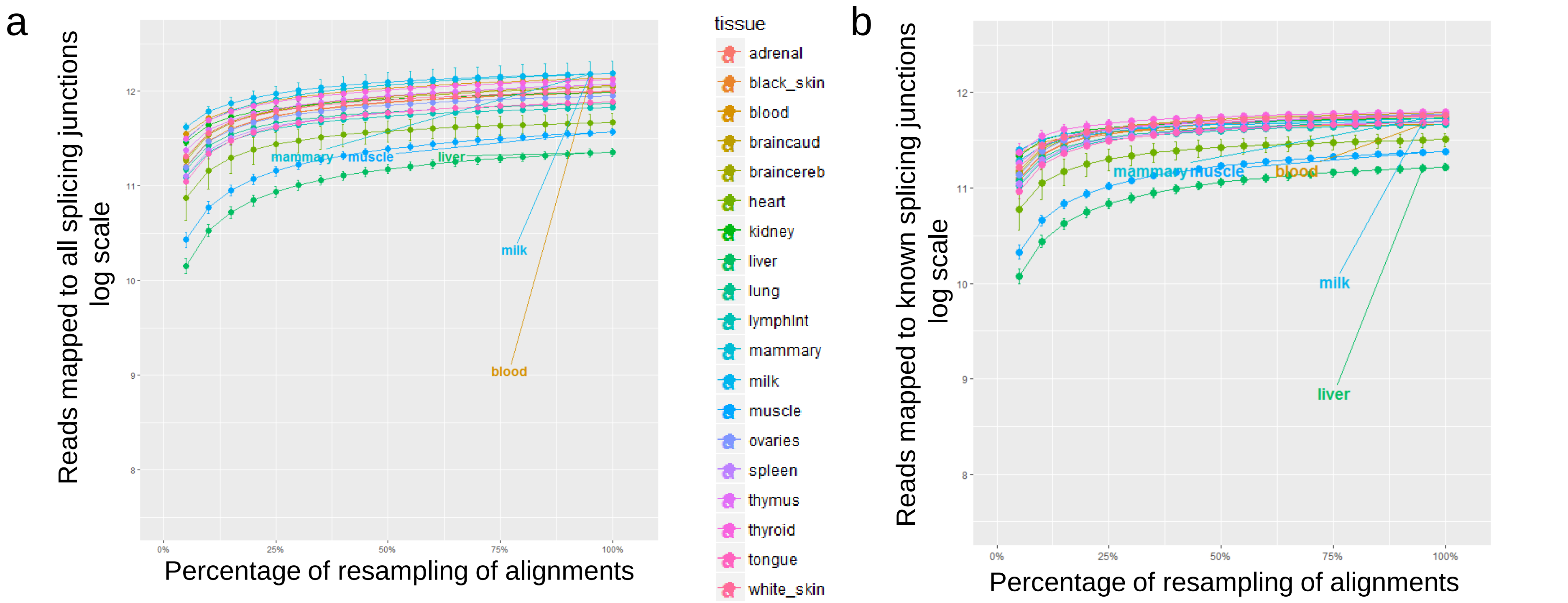

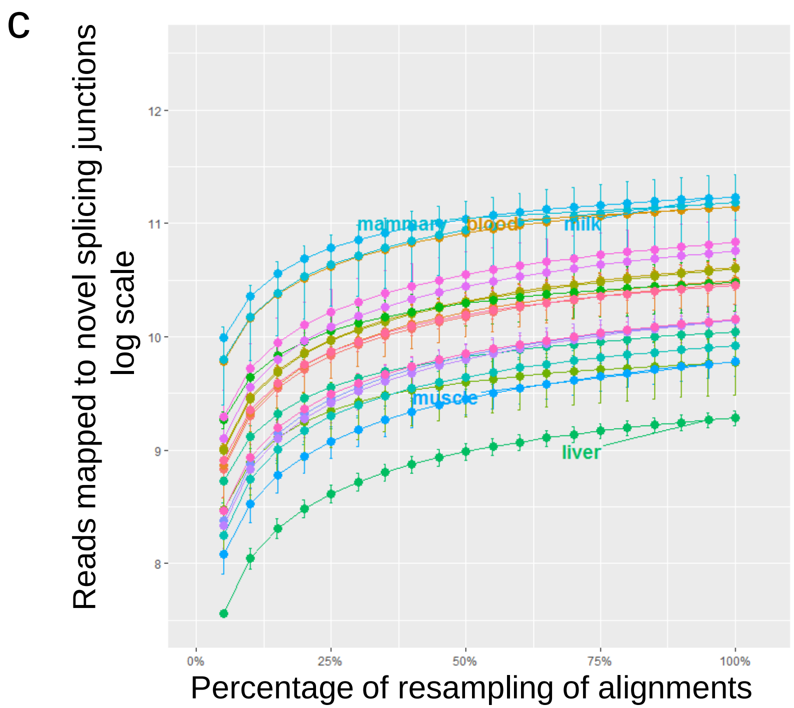

Figure S2. Splicing junction saturation analysis for each tissue type for a) all splicing junctions, b) known splicing junctions and c) novel splicing junctions. Each plot shows the mean reads mapped, with standard error bars, to splicing junctions (y-axis) for subsets of the total reads mapped (x-axis), as resampled by RSeQC (2). braincaud: brain caudal lobe. braincereb: brain cerebellum. lymphInt: Intestinal lymph node.

[2]: Wang, L., Wang, S., and Li, W. (2012). RSeQC: quality control of RNA-seq experiments. Bioinformatics 28(16), 2184-2185.

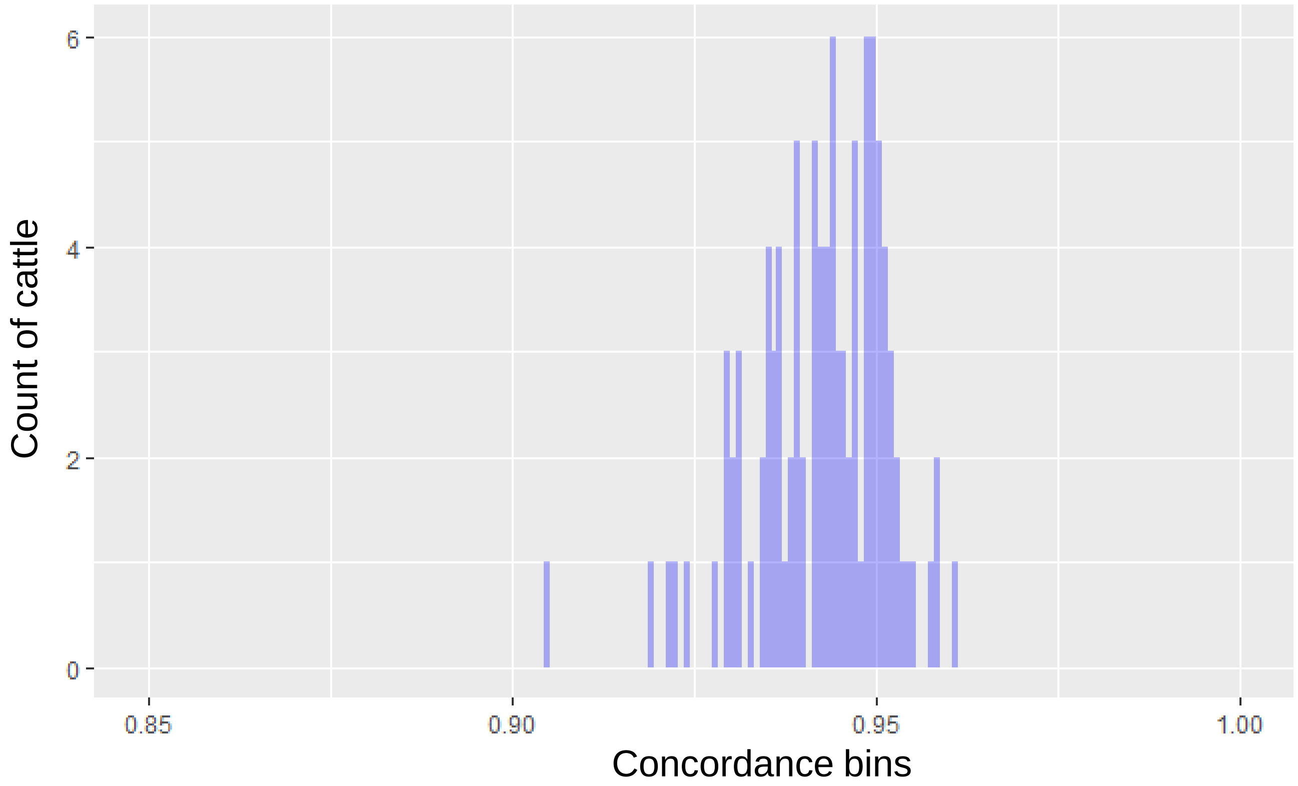

Figure S3. The distribution of concordance between imputed and RNA sequence genotypes. Where imputed genotypes are from the 1000 bull genomes project and the RNA sequence genotypes are from white blood cell RNA sequence data (Table 1). The average concordance is 0.943.

[3] Daetwyler, H.D., Capitan, A., Pausch, H., Stothard, P., Van Binsbergen, R., Brøndum, R.F., et al. (2014). Whole-genome sequencing of 234 bulls facilitates mapping of monogenic and complex traits in cattle. Nature genetics 46(8), 858-865.

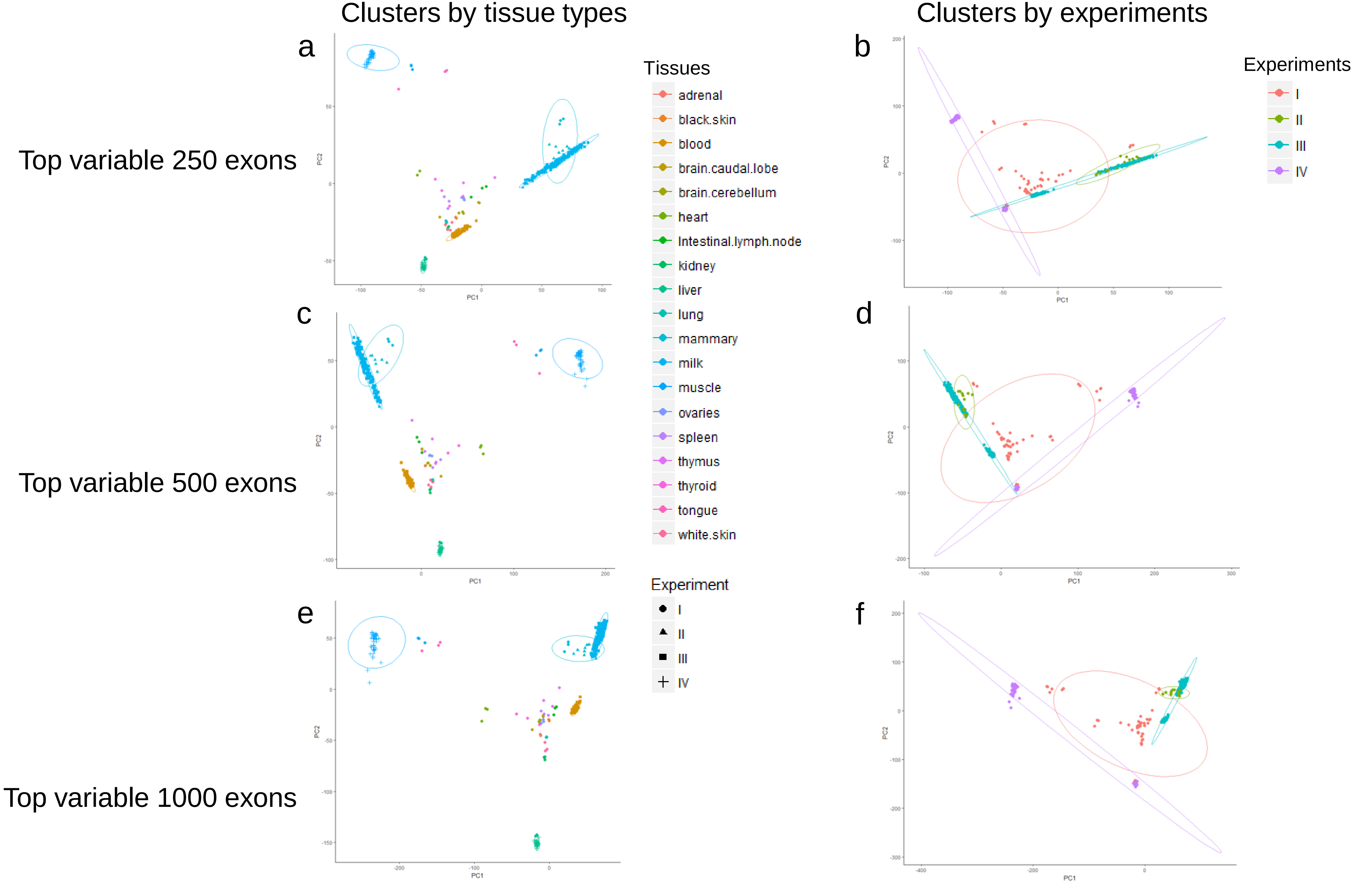

Figure S4. Sample principal components clustering based on exon expression. The principal components analysis was based on the top 250 (a,b), the top 500 (c,d) and the top 1000 (e,f) exons with the most variable expression levels across tissues samples. The confidence interval was set to 0.95 to which ellipses were drawn based on data categorized by tissue types (a,c,e) or by experiments (b,d,f). Separated ellipses indicated independence of clusters while overlapped ellipses indicated dependence of clusters.

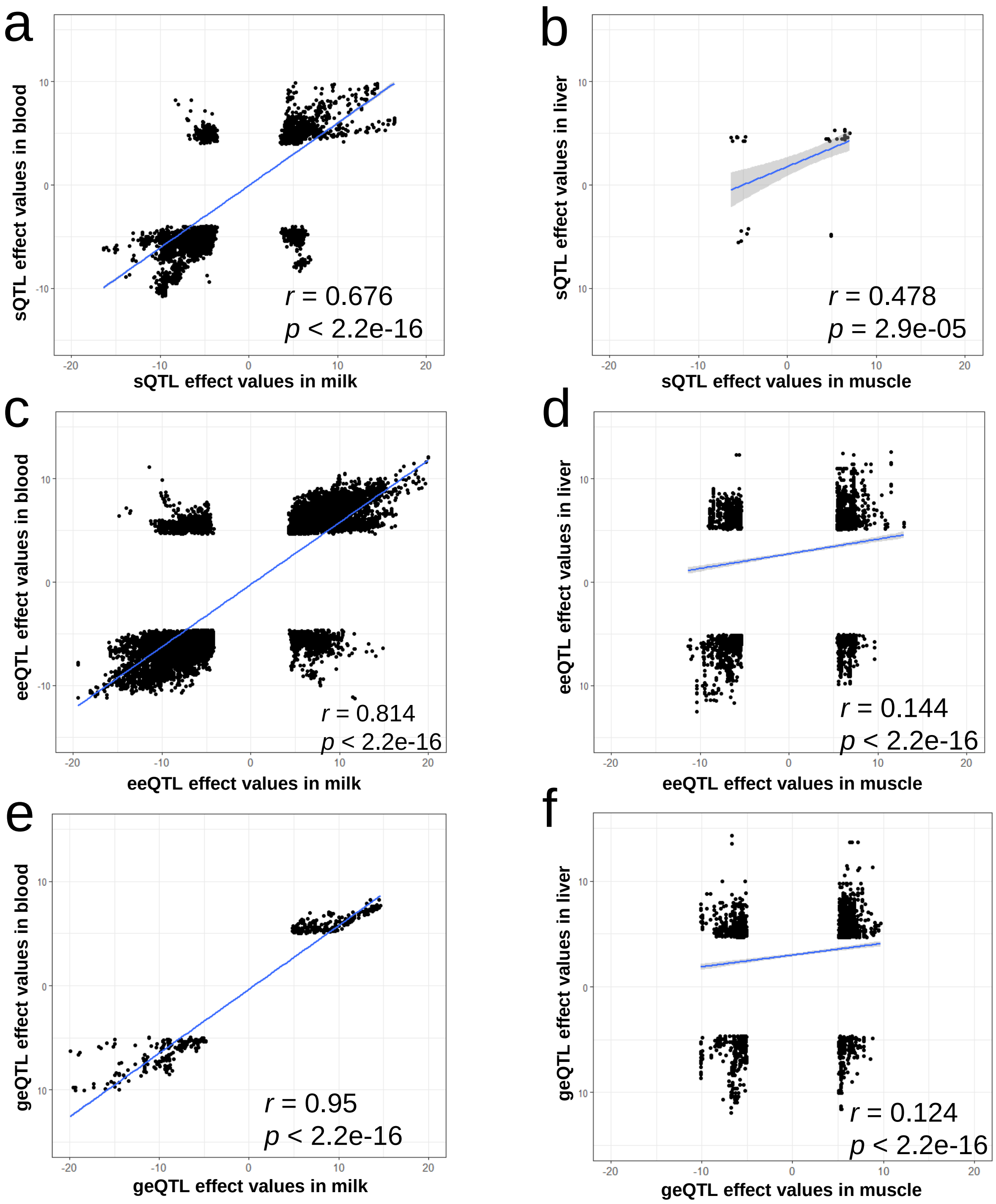

Figure S5. Correlation of sQTLs (splicing quantitative trait loci), eeQTLs (exon expression QTLs) and geQTLs (gene expression QTLs) effects (t values) between tissues. Tested tissues pairs included blood and milk (**a**, **c**, **e**) and liver and muscle (**b**, **d**, **f**). The best expression QTLs (the smallest p value) of each type were selected to for correlation analyse.

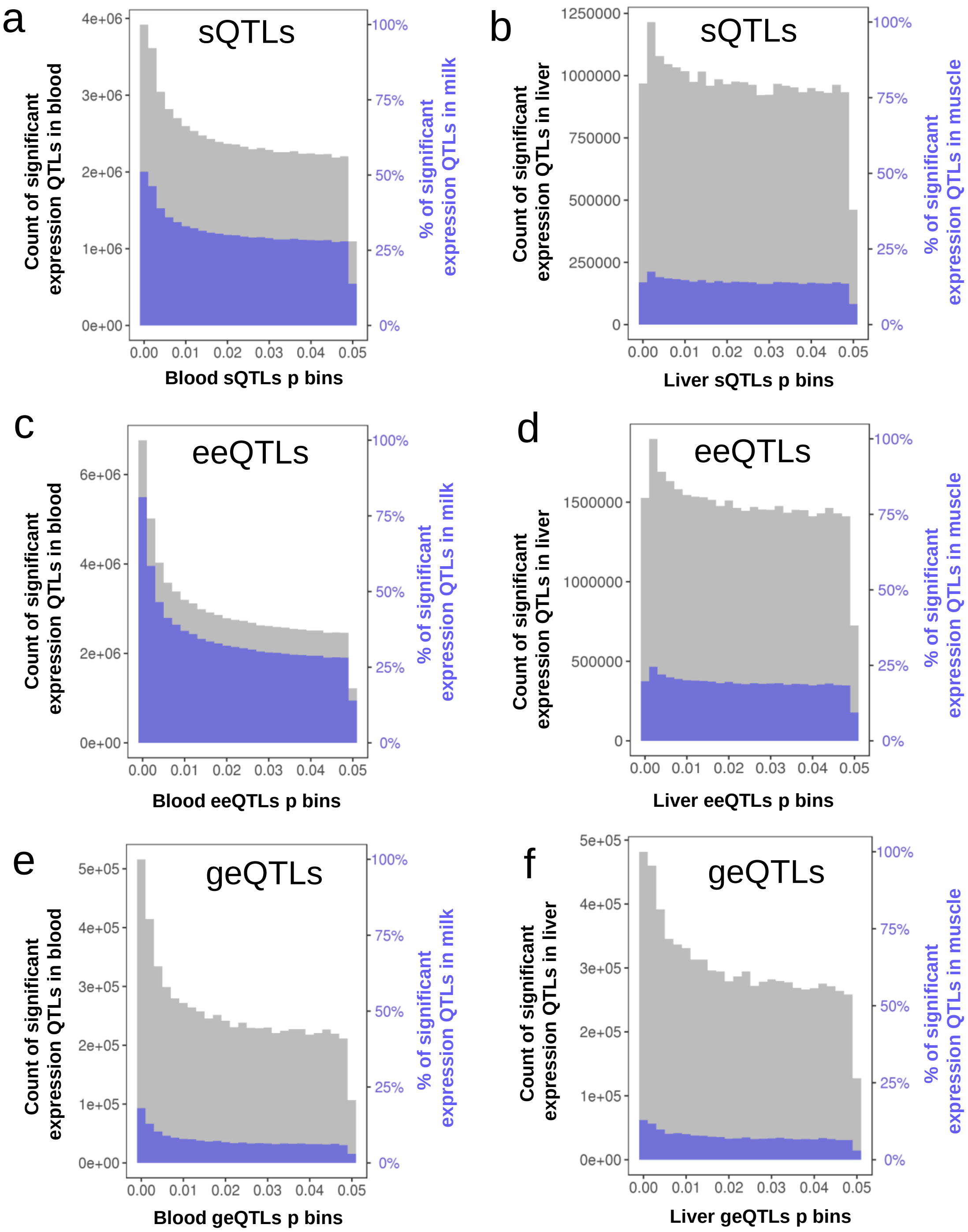

Figure S6. Sharing of sQTLs (splicing quantitative trait loci) , eeQTLs (exon expression QTLs) and geQTLs (gene expression QTLs) in paired tissues at $p<0.05$ level. The grey bars and the grey left y-axes in each panel corresponded to the frequency of SNPs at various significance bins up to $p<0.05$ in white blood cells (a, c, e) and in liver (b, d, f). In panel a, c, e, the blue bars and the blue right y-axes corresponded to the proportion of the expression QTLs in white blood cells that are also expression QTLs in milk cells in the same significance level ($p<0.05$). In panel b, d, f, the blue bars and the blue right y-axes corresponded to the proportion of the expression QTLs in liver that are also expression QTLs in muscle.

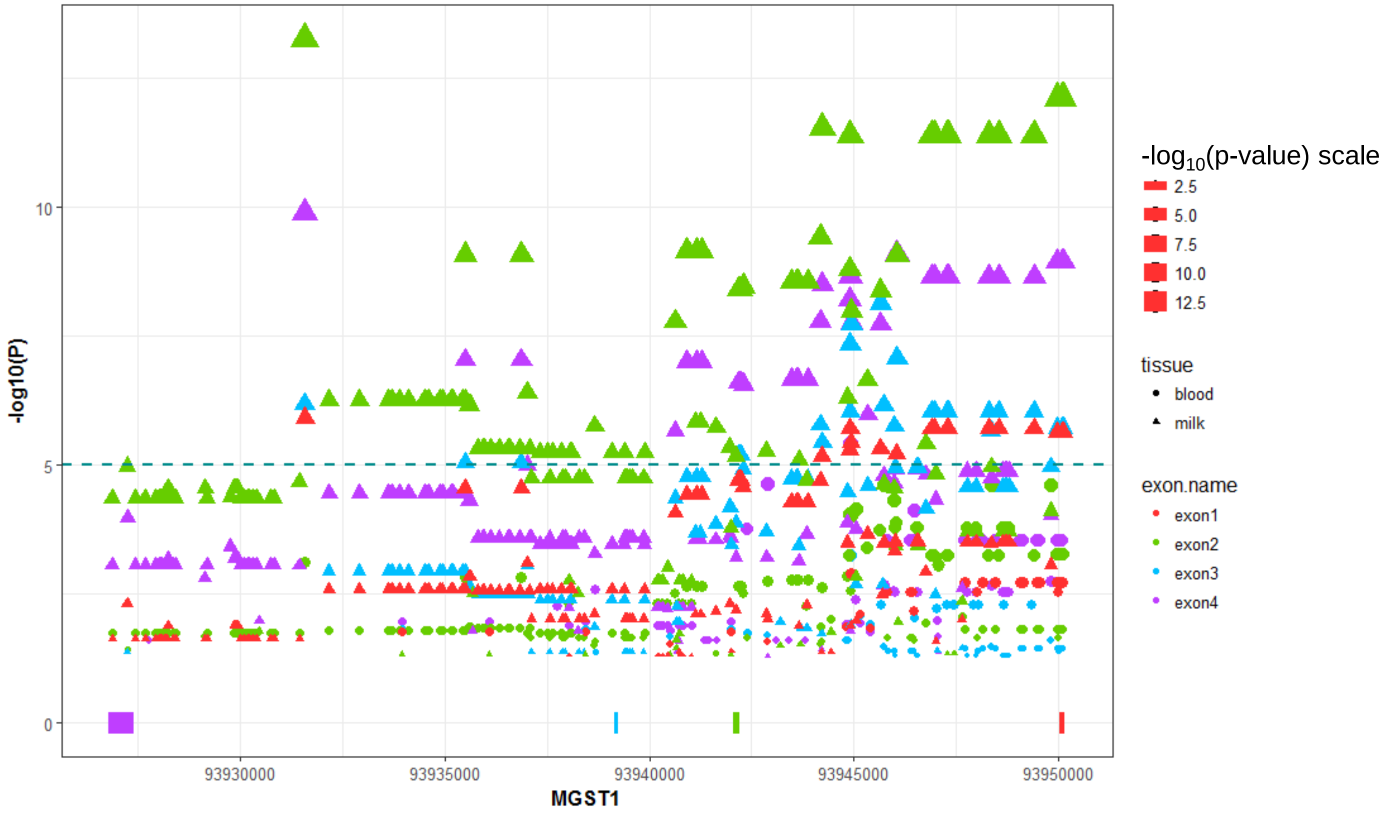

Figure S7. Manhattan plot of eeQTLs for the MGST1 gene. eeQTL were coloured based on different exons within *MGST1*.
